# Vertical Magnetic Separation of Circulating Tumor Cells for Somatic Genomic-Alteration Analysis in Lung Cancer Patients

**DOI:** 10.1038/srep37392

**Published:** 2016-11-28

**Authors:** Chang Eun Yoo, Jong-Myeon Park, Hui-Sung Moon, Je-Gun Joung, Dae-Soon Son, Hyo-Jeong Jeon, Yeon Jeong Kim, Kyung-Yeon Han, Jong-Mu Sun, Keunchil Park, Donghyun Park, Woong-Yang Park

**Affiliations:** 1 Samsung Biomedical Research Institute (SBRI), Samsung Advanced Institute of Technology (SAIT), Samsung Electronics Co. Ltd., Seoul 06351, Korea; 2 Samsung Genome Institute (SGI), Samsung Medical Center (SMC), Seoul 06351, Korea; 3 Samsung Electronics Co. Ltd., Suwon, 16677, Korea; 4 Department of Medicine, Sungkyunkwan University School of Medicine, Suwon 16416, Korea; 5 Department of Molecular Cell Biology, Sungkyunkwan University School of Medicine, Suwon 16419, Korea

## Abstract

Efficient isolation and genetic analysis of circulating tumor cells (CTCs) from cancer patients’ blood is a critical step for clinical applications using CTCs. Here, we report a novel CTC-isolation method and subsequent genetic analysis. CTCs from the blood were complexed with magnetic beads coated with antibodies against the epithelial cell adhesion molecule (EpCAM) and separated vertically on a density-gradient medium in a modified well-plate. The recovery rate of model CTCs was reasonable and the cell purity was enhanced dramatically when compared to those parameters obtained using a conventional magnetic isolation method. CTCs were recovered from an increased number of patient samples using our magnetic system vs. the FDA-approved CellSearch system (100% vs. 33%, respectively). In 8 of 13 cases, targeted deep sequencing analysis of CTCs revealed private point mutations present in CTCs but not in matched tumor samples and white blood cells (WBCs), which was also validated by droplet digital PCR. Copy-number alterations in CTCs were also observed in the corresponding tumor tissues for some patients. In this report, we showed that CTCs isolated by the EpCAM-based method had complex and diverse genetic features that were similar to those of tumor samples in some, but not all, cases.

Circulating tumor cells (CTCs) are rare tumor cells that disseminate from primary tumors or metastatic sites and then enter the bloodstream, and are believed to play a critical role in metastasis. The biological significance of CTCs in cancer originates from their potential role in metastasis, which accounts for over 90% of cancer-related deaths^1^,^2^,^3^. CTCs can serve as a noninvasive and repeatedly accessible source of tumor material that is not readily available from conventional biopsy approaches; thus, detection and characterization of CTCs can be considered as a “liquid biopsy” used to monitor disease progression and define the tumor at the molecular level through simple blood sampling in the near future^4^,^5^,^6^.

For CTCs to be utilized as valid materials for a liquid biopsy, their roles must be fully validated in specific clinical settings. Although the number of CTCs has been correlated with overall and progression-free survival (OS and PFS, respectively) in metastatic patients with different types of cancers^4^, the molecular characterization of CTCs could provide a more effective tool for personalized therapy than enumeration^7^. Thus, it is anticipated that both enumeration and characterization of the biomolecular features of CTCs should be assessed for clinical diagnosis when using CTCs in liquid biopsies.

Several techniques have recently been developed to efficiently isolate rare CTCs from peripheral blood^8^. The FDA-approved CellSearch system is based on immunomagnetic separation, which is used to target a specific antigen by using an antibody that is coupled to magnetic beads with subsequent separation of the antigen-antibody complex via exposure to a magnetic field. The isolation and detection of CTCs by the CellSearch system is effective enough to show prognostic significance, through assessing the number of detected CTCs in metastatic breast, colorectal, and prostate cancer^9^,^10^,^11^. However, the molecular characterization of these isolated CTCs is very challenging as the number of simultaneously isolated white blood cells (WBCs) is extremely high compared to that of isolated CTCs (~10,000 WBCs per test), which is especially problematic for next-generation sequencing^12^.

For the molecular analysis of CTCs, contaminating WBCs can be minimized by sorting and collecting isolated CTCs at the single- or multiple-cell level, using a micromanipulator, fluorescence-activated cell sorting (FACS), or dielectrophoresis^13^,^14^,^15^. These techniques have led to success in analyzing the genetic features of pure CTCs, thereby minimizing interference from WBCs. To sort and collect high numbers of CTCs, it is necessary to decrease contamination by WBCs during the isolation step as much as possible because this contamination may require additional purification steps, such as sorting and cell collection, which lead to lower yields of isolated CTCs.

Because of the rarity and heterogeneity of CTCs, the detailed genetic analysis of CTCs is still in its infancy^14^,^15^. However, some reports have presented genetic analyses of isolated and purified CTCs^7^,^12^,^13^,^14^,^15^,^16^,^17^,^18^. Whereas some studies have focused on detecting point mutations existing in matched tumor specimens^7^,^12^,^13^,^14^,^16^,^17^, others have analyzed copy-number alterations (CNAs) in CTCs compared with matched tumor specimens^14^,^18^. Genetic features of CTCs matching tumor specimens were observed in some cases, but exclusive genetic features of CTCs, which were different from those of tumor samples, were also reported. Considering the genetic complexities and aforementioned features of CTCs themselves, it is desirable to detect mutations and compare CNAs between CTCs and tumor samples, simultaneously, to describe the genetic features of CTCs^14^.

In the present study, we conducted isolation and genetic analysis of CTCs for the purpose of enumeration and characterizing their genetic features. The overall isolation and collection process is described in Fig. 1. The isolation technique was based on the high-density and magnetic properties of CTC-magnetic microbead complexes, which were reported previously^19^,^20^,^21^,^22^. CTC-magnetic microbead complexes could be separated vertically in modified well-plates via magnetic force, due to their high density and magnetic properties (Fig. 1A). The recovery rate and purity of CTCs were confirmed using model cell lines and through comparing the number of CTC isolated using this technique with versus the CellSearch system. After removing excess magnetic beads and retrieving isolated CTC-magnetic bead complexes using a 3D-microfilter (Fig. 1B)^23^, pure CTC-magnetic bead complexes were collected using a micromanipulator (Fig. 1C). After whole-genome amplification (WGA) of collected pure CTC-magnetic bead complexes, targeted deep sequencing and whole-genome sequencing were performed to compare point mutations and CNAs in CTCs with those of matched tumor samples and WBCs.

## Results

### Isolation of model CTCs by vertical magnetic separation

We previously reported the development of CTC-isolation methods, based on changes in physical properties such as size and following CTC complexation with magnetic beads^19^,^20^,^21^,^22^. CTC-magnetic bead complexes could be isolated with high yield and purity by centrifugation through density-gradient media. This high purity was due to differences in density between CTC-magnetic bead complexes and WBCs, which led to clear differentiation in density-gradient medium. Therefore, we hypothesized that the high yield and purity of CTCs by magnetic separation could be achieved using the same separation principle, but with centrifugal force replaced with vertical magnetic force.

**Figure 1:**
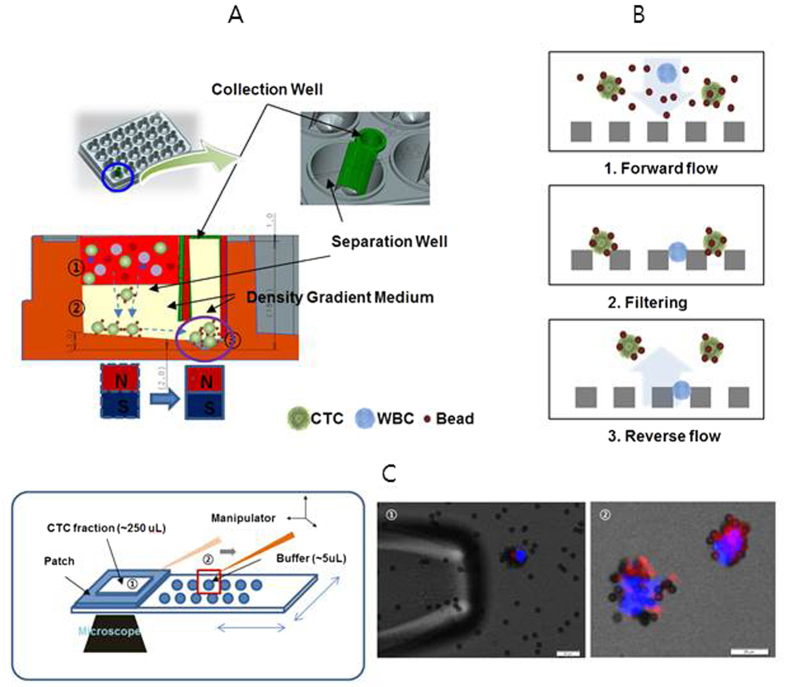
Schematic illustration of the procedure used to isolate and collect CTCs. (**A**) Isolation of CTC by vertical magnetic separation: ① Loading of blood containing CTC-magnetic beads complexes and WBCs onto density-gradient medium ② Separation of CTC-magnetic bead complexes in density-gradient medium using magnetic force ③ Collection of the separated CTC-magnetic bead complexes by moving the magnet from the separation chamber to the collection chamber. (**B**) Retrieval of isolated CTC on a 3D-microfilter: Removal of excess beads and filtering of the CTC-magnetic beads complexes by forward flow (1, 2) and retrieval of the filtered CTC-magnetic beads complexes by reverse flow (3). (**C**) Collection of retrieved CTC-magnetic beads complexes using a micromanipulator.

Thus, we developed a modified well-plate to prove this concept, as schematically described in Fig. 1A and Supplementary Fig. S1. Each well consisted of a separation and collection well, formed by fitting of an insert. Both wells were connected by openings at the bottom. The separation and collection process was performed as follows. A magnet was first placed under the separation well. After filling the wells with density-gradient media (90% Percoll), processed blood samples containing CTC-magnetic bead complexes were loaded onto the density-gradient media in the separation well. After vertical separation of CTC-magnetic bead complexes by magnetic force, CTC-magnetic bead complexes could be moved to the collection well by placing the magnet under the collection well. After collecting CTC-magnetic bead complexes using a micropipette, they were injected into a microchip filter for enumeration^19^,^20^,^21^,^22^,^23^.

**Figure 2:**
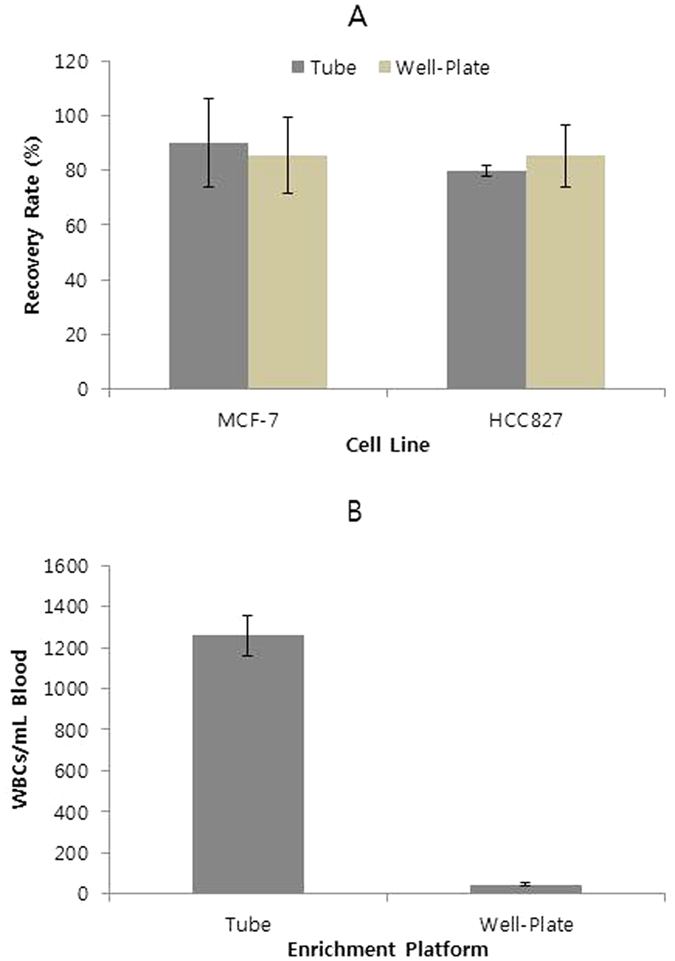
(**A**) Recovery rates from whole blood spiked with ~10 cells/ml, using 2 different cell lines. (**B**) Number of WBCs recovered by horizontal magnetic separation in tubes, or by vertical magnetic separation in well-plates (n = 3).

To test the performance of the well-plate platform in isolating CTCs from plasma-depleted blood samples, we measured the recovery rate using a spiked model of CTCs involving MCF-7 and HCC827 cells added to plasma-depleted blood samples. The purity (number of WBCs recovered simultaneously with CTCs) using this platform was then compared with the results obtained by general tube-based magnetic separation. As shown in Fig. 2A, the recovery rates for model CTCs were similar for both methods, indicating that our method performed well. Notably, the well-plate platform based on vertical magnetic separation using density-gradient media dramatically improved the purity of CTCs. As shown in Fig. 2B, this technique decreased the number of contaminating WBCs to <100 per mL blood, while maintaining the recovery rate. This value was lower by approximately 2 orders of magnitude, compared to results obtained by general magnetic-separation methods and our previous results obtained using centrifugal force in a tube and disc^19^,^20^. This result suggested that selective sedimentation of CTC-magnetic bead complexes through density-gradient media using magnetic force performed similarly to centrifugal force. Therefore, we achieved a high recovery rate and enhanced purity by using a simple well-plate and magnet without other equipment, such as a specially designed centrifuge.

### Enumeration of CTCs from patient blood samples

**Figure 3:**
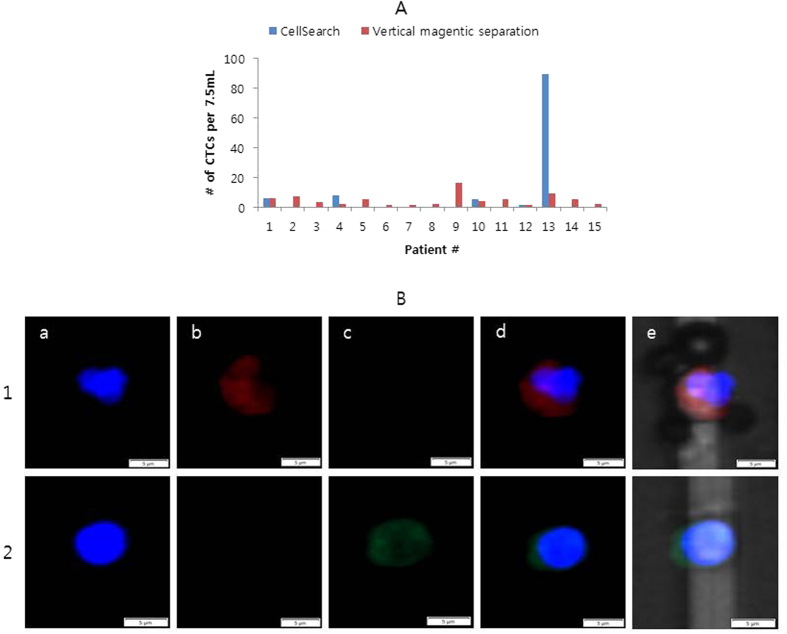
CTC isolation from cancer patients. (**A**) Comparison of the number of CTCs isolated using the vertical magnetic separation in a well-plate and by using the CellSearch System^21^. (**B**) Stained image of CTCs (line 1) and WBC (line 2) from patient 2 using vertical magnetic separation in a well-plate. a; DAPI; b: Cytokeratin (CK); c: CD45, d: Merged image of the 3 images, e: Merged image including the bright-field image.

We then compared our vertical magnetic-separation method to the CellSearch System using samples from patients with lung cancer (Fig. 3 and Supplementary Table S1). CTC staining and definition was performed as described previously^21^. This study was conducted simultaneously with our previous study^21^ during the course of developing diverse isolation platforms, and the CellSearch system was used as a reference platform in both cases. CTCs were isolated to compare platforms, using samples from the same cohort. The CTC-enumeration data obtained using the well-plate platform was compared with the CellSearch data from our previous report^21^, as a reference. CTCs were identified in 5 of 15 patients using the CellSearch system (mean, 7.0; range, 0 to 89; median, 0 per 7.5 mL of blood). By using the well-plate platform, CTCs were identified in all 15 patients (mean, 5.0; range, 1 to 16; median, 4 per 7.5 mL of blood).

The identification of CTCs in more patient samples using vertical magnetic separation might imply that this method is more sensitive and suitable isolation method for patients with a small number of CTCs that have appropriate expression levels of EpCAM.

### Pure CTC collection

Although a highly pure fraction of CTCs could be isolated by vertical magnetic separation, many WBCs remained, which could interfere with genetic analysis. In addition, excess magnetic beads were also present. Although it is feasible to detect a few specific mutations in isolated CTCs with this level of contamination^20^, it is desirable to use a pure CTC fraction for genetic profiling across broad genomic regions.

Excess beads could be removed by filtration and retrieval (Fig. 1B), as described previously^23^. The gap size of the microfilter was 6 μm, which is sufficient for passage of magnetic beads (4.8 μm in diameter) and retaining CTC-magnetic bead complexes. After filtration, CTC-magnetic bead complexes could be recovered by reverse flow using a filter. Most magnetic beads (>99.99%) were removed, and the recovery rate of CTC-magnetic bead complexes was approximately 80%. Subsequently, the recovered fraction of CTC-magnetic bead complexes was applied to specially designed slide glass^24^, and pure CTC-magnetic bead complexes were identified by microscopy and collected using a micromanipulator (Fig. 1C).

We isolated CTCs from 28 patient samples, and the results are shown in Supplementary Table S2. The average percent of collected CTCs relative to the total number of CTCs identified for each sample was 82 (±19)%. This percentage was very high compared to that of other studies, based on a combination of CellSearch isolation and micromanipulation^18^ or immune-enrichment and FACS^17^. This high percentage could be achieved by minimizing contaminants such as WBCs and excess magnetic beads, which could interfere with identifying and isolating CTCs.

### Targeted deep sequencing

Amplified DNA from CTCs and DNA from tumor samples were sequenced using 2 different platforms. We profiled genetic variations in 25 tumor samples (Supplementary Table S2) using targeted deep sequencing based on solution hybrid selection, as this method has been successfully employed to detect point mutations present at low variant allele frequencies. Due to the poor uniformity of whole genome-amplified samples, amplified DNA from CTCs was sequenced using PCR-based target enrichment (AmpliSeq^TM^) and showed a greater enrichment power than the hybrid capture-based method. Because the target regions for the PCR-based method represented a subset of targets studied using the hybrid capture-based method, we were able to compare genetic alterations in the common target regions. We analyzed CTCs from 25 patients (3 CTC-positive samples were excluded because preparation of the library failed) using targeted sequencing, based on PCR-based target enrichment.

To select appropriate samples with enough uniformity for whole-genome amplification, we calculated the proportion of targeted bases with a coverage depth greater than 100× for all targeted bases (22,234 bases) for each patient sample and defined it as selection criterion in the case that it was more than 75%. By removing samples unfulfilled with this criterion, we selected 16 of 25 patient samples for point-mutation analysis. These proportions and other sequencing-performance data for each patient sample are listed in Supplementary Table S2. The major factor that influenced this proportion was the number of CTCs to be sequenced. For samples containing greater than or equal to five CTCs, 10 of 12 fulfilled the selection criterion. In contrast, only 6 of 13 samples fulfilled the selection criterion when the number of CTCs was less than 5.

We then performed PCR-based sequencing of 13 matched WBCs for each selected patient sample; 3 samples were omitted for which matched WBCs were not prepared. For WBCs, extracted gDNA was directly used for sequencing. The sequencing performance of the selected CTCs and WBCs are summarized in Table 1. Sequencing parameters such as mean read length, mean mapped reads per sample, and mean amplicon read depth were not different between the CTCs and WBCs (p > 0.05). Although the proportion of positions with a coverage depth greater than 100× was different between the CTCs and WBCs (p < 0.05); this difference might have been due to inevitable limitations associated with WGA.

### Point mutation analysis

Mutation analysis was performed for 13 patients from whom CTCs, WBCs, and tumor samples were collected and sequenced. Despite the use of 2 different sequencing platforms, we could compare genetic alterations from different samples in common target regions, as described above. For variants called by each sequencing platform, we first selected variants with at least 100× read depth and a variant allele frequency (VAF) greater than 3% in the common target regions and selected additional variants that satisfied the selection criteria in common target regions, but were not called by analyzing the raw read counts.

After germline single-nucleotide polymorphisms (SNPs) were removed from the analysis, only nonsynonymous substitutions previously reported in the COSMIC cancer database were considered. Namely, variants in CTCs and tumor samples were called point mutations if a variant had been described as a COSMIC mutation, if the variant shared the same amino acid residue as a COSMIC mutation, and/or if the variant was not a known SNP and not present in any WBCs^25^. Although detected in tumor samples, frameshift mutations in CTCs and WBCs were excluded from analysis due to the known limitations of ion-semiconductor sequencing, in order to accurately detect frameshift mutations^17^.

The number of patients for which point mutations were observed was 8 out of 13 for CTCs and 11 out of 13 for tumor samples. The number of patients in which point mutations were observed in both CTCs and tumor samples was 6 out of 13, and the list of point mutations is summarized in Table 2. A list of all point mutations in the CTCs and tumor samples is summarized in Supplementary Table S3. The total number of point mutations observed was 14 in CTCs and 21 in tumor samples, and the average VAF of the detected mutations was 8% for CTCs and 24% for tumor samples. As shown in Table 2, the point mutations observed in CTCs did not include all mutations present in tumor samples, indicating that CTCs had genetic features distinctive from those of tissue samples.

Droplet digital PCR (ddPCR) was performed to validate 13 mutations detected in CTCs by targeted deep sequencing. Although 4 mutations were not detected by ddPCR, 9 out of 13 mutations observed in CTCs by targeted sequencing were also detected with similar VAFs by ddPCR (Table 3 and Supplementary Table S3). Therefore, the possibility of artifacts could be excluded for the mutations detected by both targeted sequencing and ddPCR.

### CNA Analysis

It was shown that most CTCs had private point mutations, meaning that CTCs had exclusive genetic features different from those of tissues. In addition to point mutations, we analyze CNAs of CTCs to determine if these features of CTCs correlate with those of tissue. To do this, CNAs of CTCs, tumor samples were analyzed for 5 of 13 patient samples, including four patients with point mutations exclusive to either CTCs or tumor tissue (patients 20, 22, 32, and 36) and one patient with point mutations in CTCs only (patient 23).

**Figure 4:**
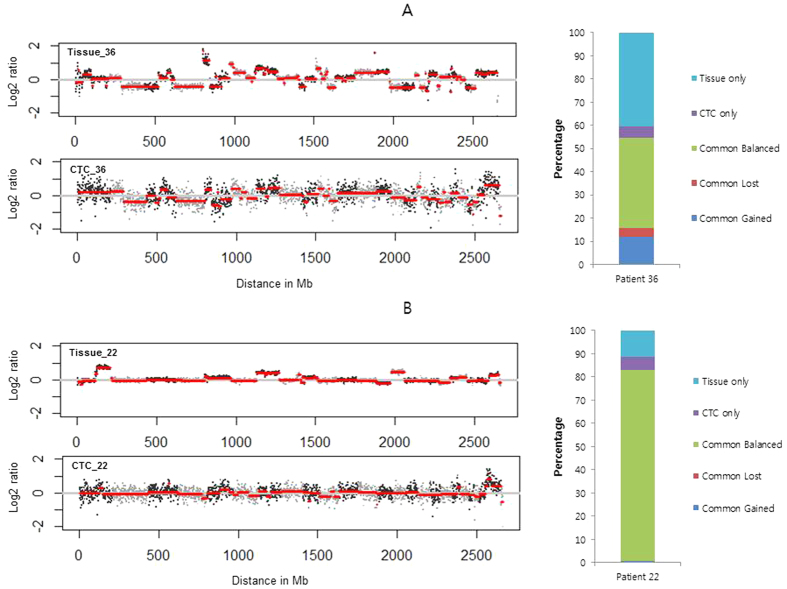
Ratio profiles of copy numbers and percentages of CNAs in CTCs and tumor samples for patient 36 (**A**) and patient 22 (**B**).

To compare CNAs between CTCs and their paired tissues, the log ratio of read counts between CTC/tissue and WBC (germline control) at every bin position for each sample was obtained by using the open-source web analytic platform, Ginkgo (http://qb.cshl.edu/ginkgo). After calculating copy-number variation by using log ratio, the CNA at each bin was compared between CTCs and their paired tissues. Representative results for 2 cases of copy-number profile and the degree of sharing CNAs among samples are described in Fig. 4.

For patient 36, the CTCs showed similar CNAs compared to the paired tissue (Fig. 4A). Detailed analysis revealed that 55% of the copy-number status in CTCs was identical to that in the tissue (39% remained balanced, 12% showed a gain, and 4% showed a loss in both sample types). Forty percent were unique to the tissues and 5% were unique to the CTCs. This analysis for patient 36 implied that some CNAs in the CTCs reflected those in matched tissue^14^. In contrast, for patient 22, CTCs showed different CNAs compared with tissue, except for a common, balanced region (Fig. 4B). Eighty-two percent of the copy-number status in CTCs and tissue remained balanced, and there were a little common gain (<1%), but no common loss. Twelve percent were unique to tissues and 6% were unique to CTCs. Although some unique alterations in both CTCs and tissue existed, the copy-number status of CTCs in patient 22 appeared similar with those of the germline samples (WBC). The CNAs of CTCs from patients 23 and 32 showed similar results compared with those observed for patient 22 (Supplementary Fig. S2). For patient 20, almost all (97%) CNAs remained commonly balanced, both in CTCs and in tissues (Supplementary Fig. S2).

## Discussion

Separation of biomaterials such as cells, proteins, and nucleic acids bound to magnetic beads is typically performed by magnetic force in the horizontal direction. Although the separation efficiency is generally high, many contaminants are separated simultaneously during the physical separation of magnetic beads. For CTC isolation using magnetic beads or particles such as CellSearch, approximately 10,000 WBCs were isolated simultaneously with rare CTCs^12^. To minimize these contaminants, it is necessary to wash separated CTC-magnetic beads complexes repeatedly. However, these repeated washings lead to the loss of target CTCs. By applying the magnetic force in the vertical direction through density-gradient medium, we were able to isolate pure CTC-magnetic bead complexes without repeated washing. The elimination of multiple wash steps might improve the rate of CTC recovery and consequently lead to identification of CTCs in more patients compared to the CellSearch platform^26^.

Although CTCs were identified in more patients using our vertical magnetic separation method than with the CellSearch system, many more CTCs were detected by the CellSearch system in some cases, especially for patient 13 (Fig. 3A). This large difference might be due to the technical characteristic of this isolation platform for low EpCAM-expressing CTCs. To overcome the density of Percoll for isolating CTC-magnetic bead complexes in well-plate platform, a minimum number of beads must be bound to CTCs. For CTCs with extremely low EpCAM expression, the small number of magnetic beads bound to CTCs was insufficient for isolating CTC-magnetic bead complexes through density-gradient medium^21^. In the case of patient 13, there might have been many low EpCAM-expressing CTCs that bound beads less than the minimum number required to overcome the density of Percoll. In such a case, CTCs cannot be isolated using the well-plate platform, but might be recovered by general magnetic separation. This discordance between CellSearch and the platform developed in this study was reported elsewhere^27^,^28^.

To overcome this technical limitation based on expression of the epithelial marker, marker-independent isolation techniques based on intrinsic CTC properties such as size and compressibility, using membrane filter or microfluidic devices, have been developed^29^,^30^,^31^. Although this loss of CTCs, which includes low-EpCAM expressing and EpCAM-negative CTCs, might represent a technical limit of vertical magnetic separation, the extremely high purity of isolated CTCs of this method might compensate for this shortcoming, especially for applications such as next-generation sequencing.

The sequencing performance of amplified DNA differs from patient to patient, displaying variable uniformity of amplified DNA due to amplification biases, such as allelic dropout^15^,^17^. Therefore, it is necessary to select appropriate samples that show reasonable sequencing performance. We selected appropriate samples with sufficient uniformity by defining the main criterion for selecting appropriate samples for point mutation analysis as the proportion of targeted bases with enough coverage (>100x) for all targeted bases, based on the sequencing data. This criterion was based on the assumption that this proportion might reflect the degree of consistency of whole-genome amplification. Referring to other studies reporting this proportion^17^, the difference between that of CTCs and WBCs was 45% (43% for CTCs vs. 88% for WBC). However, in this study, the difference was only 7% (Table 1), suggesting that the comparison between sequencing results from matched CTCs and WBCs was much more compatible than previously reported.

Some interesting aspects of the point mutations were observed in the CTCs and tumor samples. First, all point mutations identified in the CTCs were so-called “private point mutations,” defined as point mutations observed only in CTCs, but not in the matching tissue. Although the degree to which CTCs existed with private point mutations was variable, similar cases were reported previously^14^,^17^. A previous study reported that most private point mutations in CTCs could be observed by ultra-deep sequencing of matched tissue and might be present in tissue at a subclonal level^14^. Consistent with a previous report, CTCs isolated by an EpCAM-based technique in this study might represent subclones of the matched tissue. This might be because there are subpopulations of cancer cells in primary tumor tissue that are more prone to becoming CTCs, and isolation of CTCs consequently results in an enrichment of such subpopulations. The isolation technique based on EpCAM expression might also enrich a particular subpopulation of CTCs. In addition, even if the selection of CTCs during the isolation process is random, analysis of a small number of cells has inherent stochastic variations. Thus, although the origin of private point mutations in CTCs is presently unclear, the phenomenon might reflect the heterogeneity of primary tumors and enrichment of a subpopulation. Second, the average VAF of point mutations in CTCs was relatively low, even though sequencing was performed for multiple collected CTCs, without WBCs. The reason might be that circulating epithelial cells (CECs), which are not cancer-related cells but express EpCAM, were included in collected CTCs^32^. The genetic features of these CECs might be similar to those of WBCs and alter those of pure CTCs, such as the VAF of point mutations. This result could also be explained by the heterogeneity of CTCs, as discussed above. In addition, these low-frequency mutations could be artifacts, potentially introduced by WGA^33^ and sequencing. To check whether mutations in CTCs were due to sequencing artifacts, we conducted ddPCR for mutations in CTCs. Nine out of 13 mutations were detected by ddPCR and the VAFs of detected mutations were similar with those obtained by targeted sequencing (Table 3 and Supplementary Table S3). Therefore, the possibility of artifacts could be excluded for the mutations detected by both targeted sequencing and ddPCR.

Considering these discussion points, the reason that clinically important point mutations such as EGFR (L858R, T790M) and PIK3CA (E545K) discovered in tumor samples could not be observed in matched CTCs could be explained. A possible technique for overcoming these mismatched results is a census-based variant calling technique for CTC genomics in the clinic^34^. Among 10 early trunk and 56 metastatic trunk mutations in the tissue, 90% and 73%, respectively, were found in CTC exomes. Based on this report, sequencing of each single cell, severe quality control, and calling algorithm must be optimized to achieve clinical utility.

Comparisons of CNAs of CTCs with those of matched tumor samples showed that correlations between CTCs and tumor samples differed from patient to patient. Of the 5 patient samples used for CNA analysis, 1 patient displayed partially similar genetic alterations between CTCs and the tumor sample (patient 36). Three patients showed few common alterations between the CTCs and tumor samples, although some unique alterations in both CTCs and tissue existed (patients 22, 23, and 32). One patient showed little alteration in the CTCs and tumor samples (patient 20). Variable correlation of CNAs between CTCs and tumor samples from patient to patient might also arise from CTC heterogeneity and contaminating circulating epithelial cells, as mentioned above. Beyond analyzing the correlations between samples, more clinically useful information could be obtained by analyzing CNAs at a single-cell level and combining the results^18^.

In conclusion, we developed a novel isolation method for CTCs and described the genetic features of the collected CTCs. CTCs complexed with magnetic beads could be isolated efficiently and with purity by vertical magnetic force in modified well-plates. This platform enabled the recovery of CTCs from a higher proportion of patient samples when compared to that of the CellSearch system. Genetic analysis of whole-genome-amplified DNA from CTCs collected by filtration and micromanipulation revealed that CTCs had exclusive features in terms of point mutations and CNA diversity. Although clinically useful genetic features observed in matched tumor samples were not observed in CTCs, simultaneous analysis of point mutations and CNAs showed complex and diverse genetic features, which were similar with or distinctive from those of the tumor samples. Further development of analytical methods for single CTCs and determining the clinical utility of characterizing distinctive genetic features of CTCs will improve the use of CTCs as clinical biomarkers and provide research tools for cancer and other complex diseases.

## Methods

### Reagents

All materials were used as received, unless otherwise noted. Magnetic microbeads (Dynabeads^®^ Pan Mouse IgG) were purchased from Invitrogen, Inc. (Grand Island, NY, USA) and the human anti-EpCAM antibody was purchased from Novus Biologicals, LLC (Littleton, CO, USA).

### Sample processing

Healthy human blood samples for model CTCs, lung cancer patient blood samples, and tumor specimens were obtained from the Samsung Medical Center (Seoul, Korea). For enumeration, whole blood was collected into a CellSave Preservative Tube (Veridex, Raritan, NJ, USA) and processed within 4 h of collection. For genetic analysis, whole blood was collected into a BD Vacutainer^®^ K2E (EDTA) tube (BD Biosciences, Franklin Lakes, NJ, USA), and processed within 4 h of collection. Tumor specimens were collected from patients with lung cancer undergoing surgical resection. The study was approved by the institutional review board at Samsung Medical Center, and all the methods were carried out in accordance with the approved guidelines. Written informed consent was obtained from all subjects.

### Well-plate fabrication

Well-plates were composed of a main body and insert. The main body had the same size as a typical well-plate, and individual wells were divided into a separation well and a collection well. The separation and collection wells could be separated by placing an insert into a given well. The main body and insert of the well-plate were constructed of molded polycarbonates. A representative image and the dimensions of the main body and insert are described in the supporting information (Supplementary Fig. S1).

### Cell culture

Cell lines were obtained from the American Type Culture Collection (Manassas, VA, USA) and maintained in DMEM (MCF-7) or RPMI 1640 (HCC827) with 10% fetal bovine serum^21^.

### CTC isolation

To isolate CTCs from cell-spiked blood, a pre-determined number of cells was added to healthy human blood^35^. The absolute concentration of CTCs was 10 cells per mL of blood. After centrifuging 3 ml of cell-spiked blood at 800 × *g* for 10 min and removing 1.5 mL plasma, 1.5 mL of 1× PBS (with 2 mM EDTA) was added to the centrifuged blood. After mixing the blood well by pipetting, 30 μL of magnetic microbeads coated with anti-EpCAM antibodies (in accordance with the manufacturer’s instruction) was added to blood. After a 1 h rotation, 1 mL of blood was carefully loaded on each of 3 separation wells filled with 1 mL of 90% Percoll; a magnet was aligned under each separation well. After 30 min, the plate was carefully arranged to align the magnet with the collection well. After 10 min, 50 μL of solution containing the collected magnetic microbeads in the collection well were aspirated 3 times repeatedly, using a micropipette.

### CTC identification

After the collected microbeads were pooled and injected into a microchip filter, identification of isolated CTCs was done based on fluorescence staining and morphology^19^,^20^,^21^,^22^,^23^. The identification criteria for the MCF-7 and HCC827 cells were the same as those reported previously^21^,^36^,^37^.

Staining of isolated CTCs from patient samples was done with DAPI (to stain nucleic acids) and staining reagents (anti-CK [phycoerythrin] (PE) and anti-CD45 [allophycocyanin] (APC)), which were acquired from Veridex. The same criteria used the with CellSearch system was applied to identify CTCs.

### CTC collection

After collection and pooling of magnetic microbeads, samples were incubated with 20 μL PE-conjugated anti-EpCAM antibody (BD Biosciences, Franklin Lakes, NJ, USA), 20 μL Alexa-488-conjugated anti-CD45 antibody (Invitrogen, Inc., Grand Island, NY, USA), and 20 μL Hoechst 33342 (10 μg/μL concentration, Sigma, St Louis, MO, USA) for 1 h^13^. The samples were injected into a microchip filter and then retrieved by reverse flow^23^. After magnetic separation, the microbeads were resuspended in 500 μL 1× PBS. After layering magnetic microbeads on a specially designed glass slide^24^, individual CTCs (Hoechst+, EpCAM+, CD45−) were isolated by fluorescence microscopy (IX81-ZDC, Olympus, Tokyo, Japan) and a micromanipulator consisting of a CellTram microinjector and a Transfer NK2 micromanipulator (both from Eppendorf, Hamburg, Germany). Individual CTC samples were pooled and frozen at −20 °C.

### DNA preparation from CTCs, tumor samples, and WBCs

An isothermal method of WGA was performed on whole-cell lysates from pooled CTCs using a RepliG Kit (Qiagen, Hilden, Germany), according to the manufacturer’s instructions. Tumor sample DNA was extracted from fresh-frozen tissue or formalin-fixed, paraffin-embedded tumor sections using QIAamp kits (Qiagen), according to the manufacturer’s instructions. Extraction of DNA from WBCs was performed using the Puregene Cell and Tissue Kit (Qiagen), according to the manufacturer’s instructions.

### Targeted sequencing and point-mutation analysis

For CTC and WBC samples, 10 ng of DNA from each sample was PCR-amplified using AmpliSeq Cancer Panel Primer Pools and the Ion AmpliSeq^TM^ Library Kit 2.0 to generate 207 multiplexed amplicons (representing 50 cancer-related genes). Sequencing was performed on an Ion Torrent Personal Genome Machine (ThermoFisher Scientific, Waltham, MA, US) using the Ion PGM 200 Sequencing Kit. Torrent Suite software was employed to analyze the read counts and quality. Variants Caller software was used to identify variants. Coverage Analysis software was used to determine the degree of target coverage. For DNA from tumor samples, we performed targeted deep sequencing as follows. After extracting genomic DNA from tissue, a SureSelect customized kit (Agilent Technologies, Santa Clara, CA, USA) was used for capturing 381 cancer-related genes. Illumina HiSeq 2500 (Illumina, San Diego, CA, USA) was used for sequencing with 100-bp, paired-end reads. The sequencing reads were aligned to the human genome reference sequence (hg19) using BWA-mem (v0.7.5), SAMTOOLS (v0.1.18), Picard (v1.93), and GATK (v3.1.1) software for sorting SAM/BAM files, duplicate marking, and local realignment. Local realignment and base recalibration were performed based on dbSNP137, Mills indels, HapMap, and Omni software. Point mutations were identified using Mutect (v1.1.4). The Mpileup command in SAMTOOLS was used to compare raw read counts between AmpliSeq^TM^ and targeted-deep sequencing results by genomic positions. Point mutations were called to have a sequencing coverage of at least 100, a variant allele frequency (VAF) of over 3%, and to not be present in any of the WBC samples sequenced.

### ddPCR experiments

To validate mutations found in CTCs, ddPCR was performed using a QX200^TM^ Droplet Digital^TM^ PCR System (BioRad, Hercules, CA, US), according to the manufacturer’s instructions and a previous report^38^. Briefly, after preparing a reaction mixture containing 10–50 ng CTC DNA with primers and fluorescent probes, we partitioned this mixture into oil droplets (~15,000) generated using a QX200 droplet generator (Bio-Rad). PCR amplification was performed within each droplet using a Veriti 96-well thermal cycler (ThermoFisher Scientific). After PCR, droplets were flowed through a QX200 droplet reader (Bio-Rad) to read the fluorescence-positive and -negative droplets from each well of the plate. Analysis of the ddPCR data was performed with QuantaSoft™ software (Bio-Rad). The sequences of PCR primers and TaqMan probes used for each mutation site are shown in Supplementary Table S4. The thermocycling conditions used were 95 °C for 10 min (1 cycle); 94 °C for 30 s, 50 °C for 30 s, and 72 °C for 40 s (40 cycles); and 98 °C for 5 min.

### Whole-genome sequencing and CNA experiments

After quantification and qualification of genomic DNA from tumor samples, WBCs, and CTCs using a Qubit^®^ 2.0 Fluorometer (ThermoFisher Scientific) and a NanoDrop (ThermoFisher Scientific), DNA was sheared using the Covaris system and libraries were constructed using the TruSeq Nano DNA Library Prep Kit (Illumina) according to the manufacturer’s protocol for preparing samples for multiplexed paired-end sequencing. Low-coverage genome sequencing was performed on an Illumina HiSeq 2500 system in 100-bp, paired-end sequencing mode.

The same alignment step and targeted sequencing data processing to convert data in BAM format to BED format was performed using the bamtobed command in bedtools (v.2.17.0). To compare CNVs of the 3 patient sample types (CTC, tissue, and WBC), the log-ratio (logR) of read counts between CTC/tissue and WBC was obtained for equal-sized bins (1 Mb) using Ginkgo (http://qb.cshl.edu/ginkgo), an object of class CNA with the outliers smoothed, and then segmentation was performed using the DNAcopy R package (http://bioconductor.org/packages/DNAcopy/).

## Additional Information

**How to cite this article**: Yoo, C. E. *et al.* Vertical Magnetic Separation of Circulating Tumor Cells for Somatic Genomic-Alteration Analysis in Lung Cancer Patients. *Sci. Rep.* **6**, 37392; doi: 10.1038/srep37392 (2016).

**Publisher’s note:** Springer Nature remains neutral with regard to jurisdictional claims in published maps and institutional affiliations.

## Supplementary Material

Supplementary Information

## Figures and Tables

**Table 1:** Sequencing performance of CTCs and WBCs for 13 selected matched samples.

Sample type	CTC WGA DNA	WBC DNA	*p*-value (2-tailed t-test)
Mean read length (base pairs)	111	110	0.66
Mean mapped reads per sample (base pairs)	411,161	368,031	0.49
Mean amplicon read depth (std. dev.)	1835(±726)	1591(±625)	0.38
Proportion with coverage >100× (%)	90	97	0.00

**Table 2:** List of point mutations identified by targeted sequencing in both CTCs and tumor samples.

Patient #	Sample	Gene	Genomic Position	Amino Acid Change	VAF (%)	Mutation Type
20	Tumor	EGFR	Chr7_55259515	L858R	17.9	Nonsynonymous
	Tumor	TP53	Chr17_7573982	T96S	12.2	Stopgain
	Tumor	TP53	Chr17_7573982	E349X	11.3	Nonsynonymous
	CTC	EGFR	Chr7_55259599	G873R	3.9	Nonsynonymous
	CTC	ATM	Chr11_108236062	Q3000*	4.3	Stopgain
	CTC	TP53	Chr17_7578260	V179M	7.6	Nonsynonymous
	CTC	SMAD4	Chr18_48584594	Q256L	4.5	Nonsynonymous
22	Tumor	EGFR	Chr7_55241722	G724S	13.8	Nonsynonymous
	CTC	EGFR	Chr7_55249081	M793I	20.0	Nonsynonymous
24	Tumor	EGFR	Chr7_55249071	T790M	16.5	Nonsynonymous
	Tumor	EGFR	Chr7_55259515	L858R	44.7	Nonsynonymous
	Tumor	SMAD4	Chr18_48593406	G386D	22.5	Nonsynonymous
	CTC	MET	Chr7_116411923	R988C	7.5	Nonsynonymous
	CTC	FGFR2	Chr10_123274803	S372F	3.3	Nonsynonymous
27	Tumor	TP53	Chr17_7577545	M246V	5.1	Nonsynonymous
	CTC	FGFR2	Chr10_123279539	G298D	5.1	Nonsynonymous
32	Tumor	KIT	Chr4_5594262	N665K	34.0	Nonsynonymous
	Tumor	TP53	Chr17_7577534	R249S	45.3	Nonsynonymous
	CTC	FGFR3	Chr4_1806270	R399C	6.1	Nonsynonymous
	CTC	TP53	Chr17_7579442	P82L	4.7	Nonsynonymous
36	Tumor	TP53	Chr17_7579415	W91X	87.1	Stopgain
	CTC	ALK	Chr2_29443646	P1191S	12.2	Nonsynonymous

**Table 3:** Comparison of variants allele frequency (VAF) for mutations in CTCs detected by targeted sequencing (VAF 1) and digital PCR (VAF 2).

Patient #	Gene	Genomic Position	Ref/Alt	VAF 1 (%)	VAF2 (%)
20	EGFR	Chr7_55259599	G873R	3.9	0.0
	ATM	Chr11_108236062	Q3000*	4.3	N.A.^a^
	**TP53**	**Chr17_7578260**	**V179M**	**7.6**	**7.5**
	SMAD4	Chr18_48584594	Q256L	4.5	0.0
22	**EGFR**	**Chr7_55249081**	**M793I**	**20.0**	**17.8**
24	**MET**	**Chr7_116411923**	**R988C**	**7.5**	**7.2**
	**FGFR2**	**Chr10_123274803**	**S372F**	**3.3**	**2.6**
27	**FGFR2**	**Chr10_123279539**	**G298D**	**5.1**	**4.5**
32	FGFR3	Chr4_1806270	R399C	6.1	1.7^b^
	**TP53**	**Chr17_7579442**	**P82L**	**4.7**	**4.1**
36	**ALK**	**Chr2_29443646**	**P1191S**	**12.2**	**12.1**

^a^Data not available because a TaqMan probe for this mutation was not prepared.

^b^Mutation also detected in matched WBCs.
