# Supplementary material for: Vertical Magnetic Separation of Circulating Tumor Cells for Somatic Genomic-Alteration Analysis in Lung Cancer Patients
Source: Sci Rep. 2016 Nov 28;6:37392. doi: 10.1038/srep37392 (PMC5124952; doi:10.1038/srep37392)
Supplement: Supplementary Information [file srep37392-s1.pdf]

Vertical Magnetic Separation of Circulating Tumor Cells and Somatic Genomic-Alteration Analysis in Lung Cancer Patients

Chang Eun Yoo[1,2#], Jong-Myeon Park[3#], Hui-Sung Moon[1,2], Je-Gun Joung[2], Dae-Soon Son[1,2], Hyo-Jeong Jeon[2], Yeon Jeong Kim[1,2], Kyung-Yeon Han[1,2], Jong-Mu Sun[4], Keunchil Park[4], Donghyun Park[1,2]*, Woong-Yang Park[2,5]*

[1]Samsung Biomedical Research Institute (SBRI), Samsung Advanced Institute of Technology (SAIT), Samsung Electronics Co. Ltd., Seoul 06351, Korea

[2] Samsung Genome Institute (SGI), Samsung Medical Center (SMC), Seoul 06351, Korea

[3] Samsung Electronics Co., Ltd., Suwon, 16677, Korea

[4] Department of Medicine, Sungkyunkwan University School of Medicine, Suwon 16416, Korea

[5] Department of Molecular Cell Biology, Sungkyunkwan University School of Medicine, Suwon 16419, Korea

[#] These authors contributed equally to this work

* To whom correspondence should be addressed.

Donghyun Park

E-mail: dh37.park@samsung.com

Tel: +82 2 3410 2954

Fax: +82 2 2148 9819

Woong-Yang Park

E-mail: woongyang.park@samsung.com

Tel: +82 2 3410 6128

Fax: +82 2 2148 9819

SUPPLEMENTARY INFORMATION

Supplementary Figures S1–2

Supplementary Tables S1-4

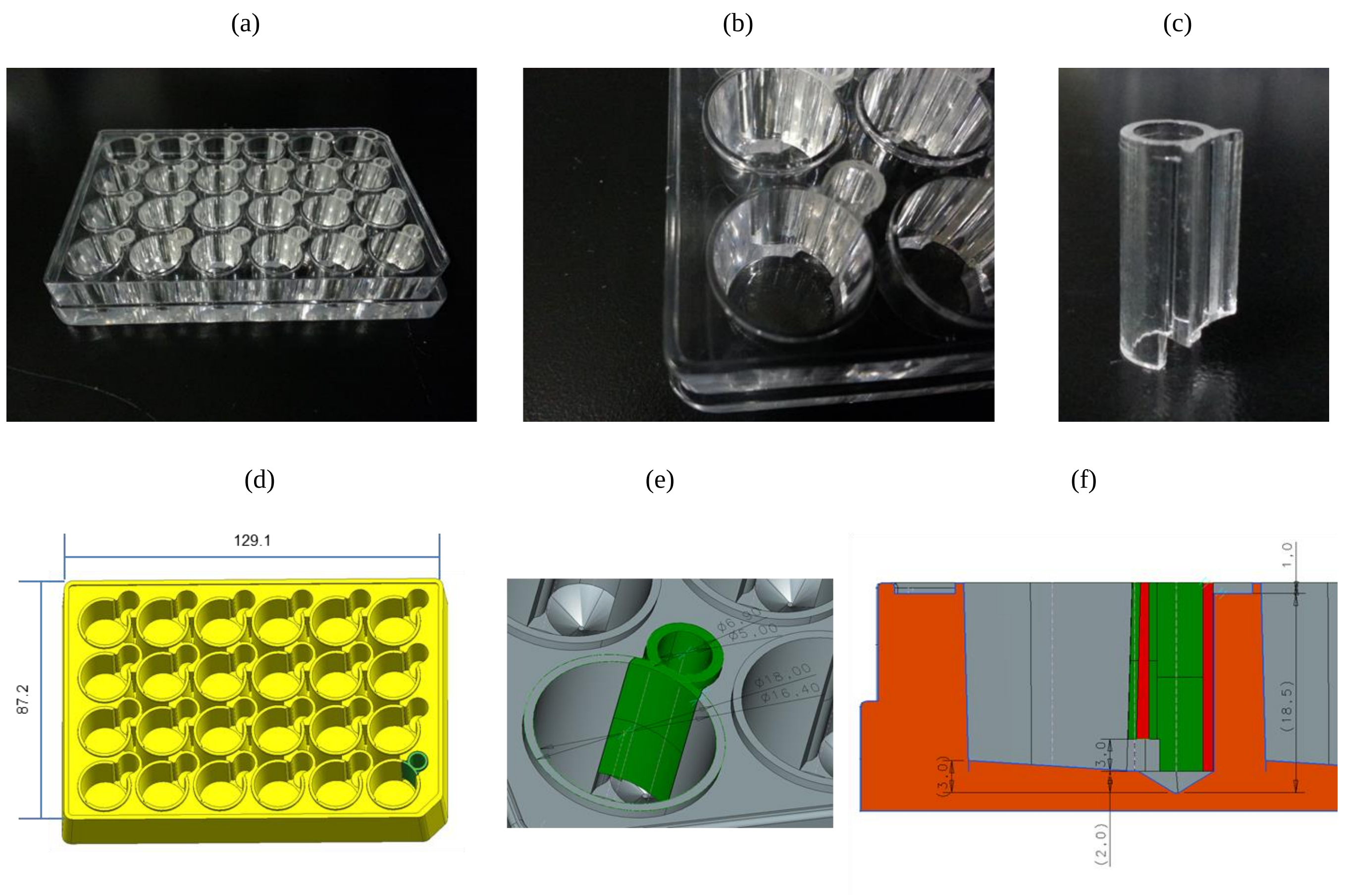

Supplementary Figure S1. Photographs, dimensions, and schematic view of the modified well-plate. (a) Plate, (b) Well, (c) Insert, (d) Plate dimensions, (e) Well dimensions, (f) Schematic view of well fitted with an insert (green)

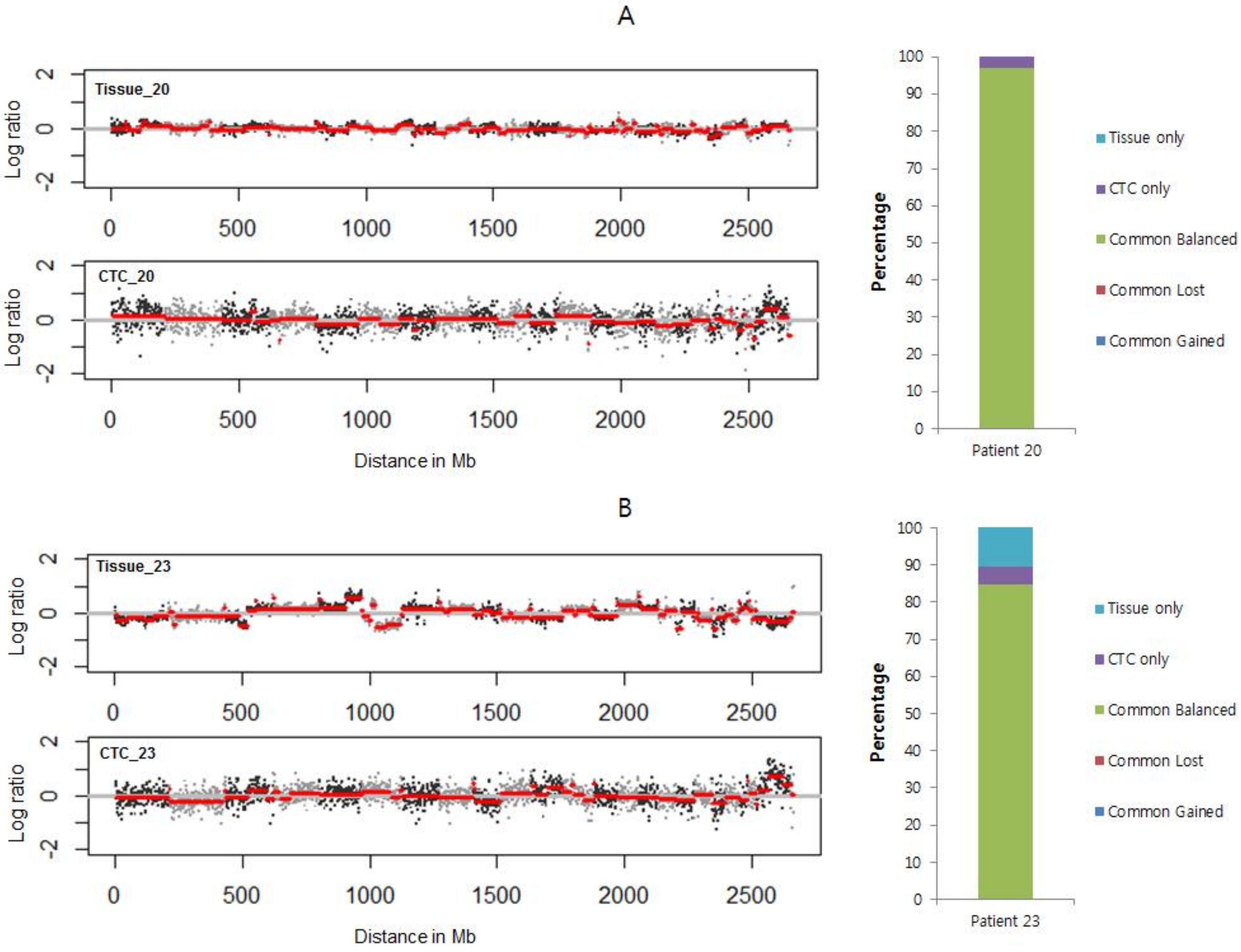
A
Tissue_20
CTC_20
Log ratio
Distance in Mb
Percentage
Patient 20
Tissue only
CTC only
Common Balanced
Common Lost
Common Gained
B
Tissue_23
CTC_23
Log ratio
Distance in Mb
Percentage
Patient 23
Tissue only
CTC only
Common Balanced
Common Lost
Common Gained

C

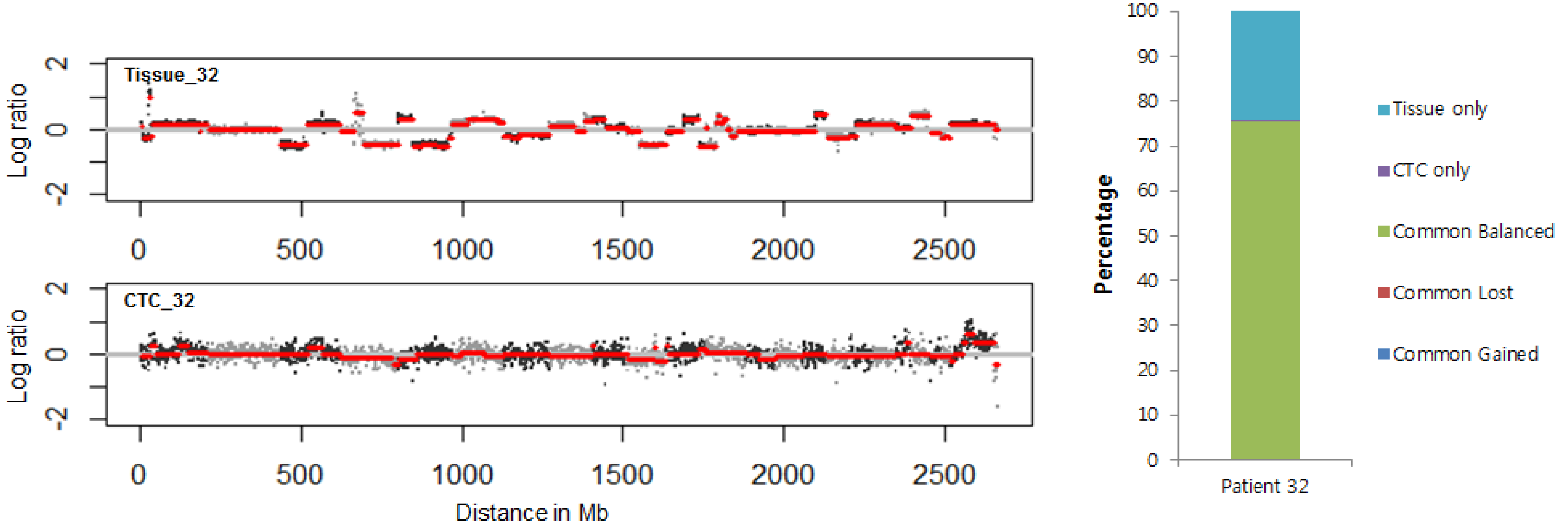

Supplementary Figure 2 Ratio profiles of copy numbers and percentage of CNAs in CTCs and tumor samples for patient 20 (A), patient 23 (B), and patient 32 (C)

Supplementary Table S1

Histological information of patient samples and comparison of the number of CTCs recovered by vertical magnetic separation and the CellSearch method[21].

Patient #	Histology	Number of CTCs per 7.5 mL	
		Vertical Magnetic Separation	CellSearch
1	ADC	6	6
2	SQ	7	0
3	ADC	3	0
4	SCLC	2	8
5	ADC	5	0
6	ADC	1	0
7	ADC	1	0
8	SQ	2	0
9	Pleomorphic ADC	16	0
10	ADC	4	5
11	ADC	5	0
12	SCLC	1	1
13	SCLC	9	89
14	SCLC	5	0
15	ADC	2	0

Supplementary Table S2

Histological information, the number of CTCs collected, and sequencing performance of collected CTCs for each patient sample

Patient #	Histology	Tumor Specimen	CTC Candidates	CTC Collected	Reads	Mean Read Length (bp)	Mapped Reads	On Target	Mean Depth	Uniformity	Base Proportion (>100×)
16	NSCLC	Frozen Tissue	8	8	Library Failed						
17	NSCLC	FFPE	2	2	261,377	112	260,129	97.25%	1,195	41.19%	49
18	NSCLC	Frozen Tissue	4	4	482,875	103	480,669	98.33%	2,011	23.70%	27
19	SCLC	Frozen Tissue	16	11	409,825	112	408,282	99.20%	1,878	80.71%	89
20	NSCLC	Frozen Tissue	18	12	303,200	105	302,163	98.80%	1,321	75.37%	87
21	NSCLC	Frozen Tissue	2	2	Library Failed						
22	NSCLC	Frozen Tissue	8	5	176,422	112	175,862	97.91%	802.4	85.23%	91
23	NSCLC	Frozen Tissue	27	20	172,049	112	171,501	97.94%	783.4	82.65%	86
24	NSCLC	Frozen Tissue	6	5	366,538	111	365,019	98.97%	1,673	80.48%	92
25	NSCLC	Frozen Tissue	8	8	478,426	102	476,669	97.76%	2,018	40.60%	57
26	NSCLC	No tumor	2	2	252,466	111	251,868	98.88%	1,143	76.23%	89
27	NSCLC	Frozen Tissue	4	4	372,147	112	371,021	98.99%	1,699	71.88%	90
28	NSCLC	Not Enough Tissue	16	10	367,705	111	366,507	98.82%	1,677	89.35%	95
29	NSCLC	FFPE	4	2	427,809	110	426,541	98.92%	1,940	86.24%	95
30	NSCLC	Frozen Tissue	5	3	467,426	113	465,517	88.87%	1,778	46.55%	60
31	NSCLC	Frozen Tissue	1	1	430,853	111	429,432	97.72%	1,944	50.50%	55
32	SCLC	Frozen Tissue	14	13	384,436	111	383,257	98.65%	1,753	91.01%	97
33	NSCLC	Not Enough Tissue	6	4	510,030	114	508,699	95.41%	2,291	20.71%	38
34	NSCLC	FFPE DNA	10	8	298,770	110	297,917	98.91%	1,343	86.58%	92

35	NSCLC	Frozen Tissue	3	3	572,204	110	570,130	97.58%	2,546	69.01%	83
36	SCLC	Frozen Tissue	12	6	678,502	111	676,223	97.54%	3,018	77.59%	93
37	NSCLC	Frozen Tissue	7	6	452,057	113	450,434	90.52%	1,874	51.63%	73
38	NSCLC	Frozen Tissue	8	7	662,850	110	660,989	94.18%	2,850	72.29%	90
39	SCLC	Not Enough Tissue	1	1	307,670	116	306,381	98.09%	1,465	25.51%	36
40	NSCLC	Not Enough Tissue	2	2	428,379	104	426,579	96.25%	1,782	36.92%	56
41	NSCLC	Frozen Tissue	3	1	349,551	112	347,914	94.46%	1,539	65.45%	79
42	NSCLC	FFPE	1	1	Library Failed						
43	NSCLC	Frozen Tissue	5	4	600,053	113	596,562	94.49%	2,589	86.50%	95
44	NSCLC	No tumor	0	0	No CTCs						
45	NSCLC	Frozen Tissue	0	0							

Supplementary Table S3

List of somatic, non-synonymous mutations identified by targeted sequencing in tumor samples and CTCs, and digital PCR in CTCs. “VAF 1” and “VAF 2” refer to the variant allele frequency measured by targeted sequencing and digital PCR, respectively.

Patient #	Sample	Gene	Genomic Position	Amino Acid Change	VAF 1(%)	VAF 2 (%)	Mutation Type	Present in COSMIC
20	Tumor	EGFR	Chr7_55259515	L858R	17.9		Nonsynonymous	COSM6224
	Tumor	TP53	Chr17_7573982	T96S	12.2		Stopgain	COSM10770
	Tumor	TP53	Chr17_7573982	E349X	11.3		Nonsynonymous	COSM707069
	CTC	EGFR	Chr7_55259599	G873R	3.9	0.0	Nonsynonymous	Unknown[a]
	CTC	ATM	Chr11_108236062	Q3000*	4.3	N.A.[b]	Stopgain	Unknown[a]
	CTC	TP53	Chr17_7578260	V179M	7.6	7.5	Nonsynonymous	COSM43779
	CTC	SMAD4	Chr18_48584594	Q256L	4.5	0.0	Nonsynonymous	Unknown[a]
22	Tumor	EGFR	Chr7_55241722	G724S	13.8		Nonsynonymous	COSM13979
	CTC	EGFR	Chr7_55249081	M793I	20.0	17.8	Nonsynonymous	COSM1716335
23	CTC	NRAS	Chr1_115258723	S17N	26.0	30.3	Nonsynonymous	COSM253332
	CTC	CSF1R	Chr5_115258723	Q311R	3.2	3.0	Nonsynonymous	Unknown[a]
24	Tumor	EGFR	Chr7_55249071	T790M	16.5		Nonsynonymous	COSM6240
	Tumor	EGFR	Chr7_55259515	L858R	44.7		Nonsynonymous	COSM6224
	Tumor	SMAD4	Chr18_48593406	G386D	22.5		Nonsynonymous	COSM1150895
	CTC	MET	Chr7_116411923	R988C	7.5	7.2	Nonsynonymous	COSM1666978
	CTC	FGFR2	Chr10_123274803	S372F	3.3	2.6	Nonsynonymous	Unknown[a]
27	Tumor	TP53	Chr17_7577545	M246V	5.1		Nonsynonymous	COSM43555
	CTC	FGFR2	Chr10_123279539	G298D	5.1	4.5	Nonsynonymous	Unknown[a]
29	Tumor	PIK3CA	Chr3_178936091	E545K	12.9		Nonsynonymous	COSM763

	Tumor	FBXW7	Chr4_153247367	R479G	22.5		Nonsynonymous	COSM22967
32	Tumor	KIT	Chr4_5594262	N665K	34.0		Nonsynonymous	COSM4413464
	Tumor	TP53	Chr17_7577534	R249S	45.3		Nonsynonymous	COSM10817
	CTC	FGFR3	Chr4_1806270	R399C	6.1	1.7[c]	Nonsynonymous	COSM296687
	CTC	TP53	Chr17_7579442	P82L	4.7	4.1	Nonsynonymous	COSM43910
34	Tumor	ATM	Chr11_108236087	R3008H	8.4		Nonsynonymous	COSM21626
	Tumor	KRAS	Chr12_25398284	G12A	18.4		Nonsynonymous	COSM522
35	Tumor	PIK3CA	Chr3_178952085	H1047R	10.8		Nonsynonymous	COSM775
	Tumor	TP53	Chr17_7577536	R249G	18.3		Nonsynonymous	COSM10668
36	Tumor	TP53	Chr17_7579415	W91X	87.1		Stopgain	COSM44191
	CTC	ALK	Chr2_29443646	P1191S	12.2	12.1	Nonsynonymous	Unknown[a]
38	CTC	STK11	Chr19_1220416	Q170R	3.8	0.3[c]	Nonsynonymous	Unknown[a]
41	Tumor	CTNNB1	Chr3_4126098	D32V	17.2		Nonsynonymous	COSM5691
43	Tumor	CDKN2A	Chr9_21971137	D74A	10.0		Nonsynonymous	Unknown[a]
	Tumor	PTEN	Chr10_89685271	F56V	34.3		Nonsynonymous	COSM5257
	Tumor	TP53	Chr17_7578239	E204X	49.0		Nonsynonymous	COSM10804

[a]. Variants with the same amino acid position, but an alternative sequence in the COSMIC database

[b]. Not available because a TaqMan probe for this mutation was not prepared

[c]. Mutation also detected in matched WBCs

Supplementary Table S4

Sequence of PCR primers and TaqMan probes used for digital PCR

Gene	Genomic Position	Ref/Alt	Forward Primer	Reverse Primer	Wild Probe (VIC)	Mutant Probe (FAM)
EGFR	Chr7_55259599	G/A	GGTGCGGAAGAGAAAGAATACCAT	CTGACCTAAAGCCACCTCCTT	ACTTTGCCTCCTTCTGC	CTTTGCCTCTTTCTGC
ATM*	Chr11_108236062	C/T	-.	-	-.	-
TP53	Chr17_7578260	C/T	CATCCAAATACTCCACACGCAAATT	GATTCCTCACTGATTGCTCTTAGGT	CATCTTATCCGAGTGGAAG	ATCTTATCCGAATGGAAG
SMAD4	Chr18_48584594	A/T	AGGACAGCAGCAGAATGGATTT	GGCTGCCTACTTTTTTCTCAACTATTTAAA	AAGTAGCTGGCTGACCAG	TAGCTGGCAGACCAG
EGFR	Chr7_55249081	G/A	CACCGTGCAGCTCATCAC	TCTTTGTGTTCCCGGACATAGTC	CAGCTCATGCCCTTCG	CAGCTCATACCCTTCG
NRAS	Chr1_115258723	C/T	TGGTTCTGGATTAGCTGGATTGTC	GTTGGAGCAGGTGGTGTTG	AGTGCGCTTTTCC	AGTGCGTTTTTCC
CSF1R	Chr5_115258723	T/C	TCCACCATGACTTTGAGGTTGAG	CTTGAACTTGAGCTCTGAGCAGAA	CCTCATCCAGGAGGTG	CTCATCCGGGAGGTG
MET	Chr7_116411923	C/T	CTCTGTTTTAAGATCTGGGCAGTGA	GCCTATCCAAATGAGGAGTGTGTAC	CATCGTAGCGAACTAA	CATCGTAGCAAACTAA
FGFR2	Chr10_123274803	G/A	TGCAGTAAATGGCTATCTCCAGGTA	CAATCTAGCGCCTGGAAGAGAAA	AGGAGATTACAGCTTCCCCAGA	AGGAGATTACAGCTTTCCCAGA
FGFR2	Chr10_123279539	C/T	GTCCTCACCTTGAGAACCTTGAG	CCCAGCCCCACATCCA	AAGAACGGCAGTAAAT	AAAAGAACGACAGTAAAT
FGFR3	Chr4_1806270	C/T	GTGGTGGCGGCTGTGA	GGAGCCCAGGCCTTTCTTG	CGCAGGCGGCAGAG	CGCAGGCAGCAGAG
TP53	Chr17_7579442	G/A	TGGGAAGGGACAGAAGATGACA	GCCCCTGCACCAGCA	CGCCGGTGTAGGAG	CCGCCAGTGTAGGAG
ALK	Chr2_29443646	G/A	CCATGAGCTCCAGCAGGAT	GCAAATTCAACCACCAGAACATTGT	ATCCCTGCCCCGGTTC	AATCCCTGTCCCGGTTC
STK11	Chr19_1220416	A/G	TGACGGCCTGGAGTACCT	CCGGCTTGATGTCCTTGTG	CAATGCCCTGGCTATG	ATGCCCCGGCTATG

* TaqMan probe for this mutation was not prepared
